# Components of the Phenylpropanoid Pathway in the Implementation of the Protective Effect of Sodium Nitroprusside on Wheat under Salinity

**DOI:** 10.3390/plants12112123

**Published:** 2023-05-26

**Authors:** Dilara Maslennikova, Sergey Ivanov, Svetlana Petrova, Guzel Burkhanova, Igor Maksimov, Oksana Lastochkina

**Affiliations:** 1 Institute of Biochemistry and Genetics UFRC RAS, 71 Pr. Oktyabrya, 450054 Ufa, Russia; 2 Ufa Institute of Chemistry UFRC RAS, 69 Pr. Oktyabrya, 450054 Ufa, Russia

**Keywords:** nitric oxide, sodium nitroprusside, *Triticum aestivum* L., phenylalanine ammonia lyase, tyrosine ammonia lyase, peroxidase, salicylic acid, lignin, salinity

## Abstract

Nitric oxide (NO) is a multifunctional, gaseous signaling molecule implicated in both physiological and protective responses to biotic and abiotic stresses, including salinity. In this work, we studied the effects of 200 µM exogenous sodium nitroprusside (SNP, a donor of NO) on the components of the phenylpropanoid pathway, such as lignin and salicylic acid (SA), and its relationship with wheat seedling growth under normal and salinity (2% NaCl) conditions. It was established that exogenous SNP contributed to the accumulation of endogenous SA and increased the level of transcription of the pathogenesis-related protein 1 (*PR1*) gene. It was found that endogenous SA played an important role in the growth-stimulating effect of SNP, as evidenced by the growth parameters. In addition, under the influence of SNP, the activation of phenylalanine ammonia lyase (PAL), tyrosine ammonia lyase (TAL), and peroxidase (POD), an increase in the level of transcription of the *TaPAL* and *TaPRX* genes, and the acceleration of lignin accumulation in the cell walls of roots were revealed. Such an increase in the barrier properties of the cell walls during the period of preadaptation played an important role in protection against salinity stress. Salinity led to significant SA accumulation and lignin deposition in the roots, strong activation of TAL, PAL, and POD, and suppression of seedling growth. Pretreatment with SNP under salinity conditions resulted in additional lignification of the root cell walls, decreased stress-induced endogenous SA generation, and lower PAL, TAL, and POD activities in comparison to untreated stressed plants. Thus, the obtained data suggested that during pretreatment with SNP, phenylpropanoid metabolism was activated (i.e., lignin and SA), which contributed to reducing the negative effects of salinity stress, as evidenced by the improved plant growth parameters.

## 1. Introduction

Wheat (*Triticum aestivum* L.) is an important cereal crop plant cultivated worldwide as a source of food [1]. More than 800 million hectares of land and 32 million hectares of agricultural land are affected by salinity stress globally [2]. By the year 2050, approximately half of the world’s arable land will be saline [2,3].

Salt stress is one of the most detrimental stresses, as it simultaneously causes ionic toxicity and osmotic and oxidative stresses [3,4,5,6,7,8,9,10,11,12,13,14]. Plants have evolved several strategies to cope with the challenge of salinity stress, including the biosynthesis of osmoprotectants, activation of the osmotic stress pathway, regulation of ion homeostasis, mediation of plant hormone signaling, regulation of cytoskeleton dynamics and cell wall composition, and the synthesis of antioxidant enzymes and compounds [7,8,9,10,11,12,13,14]. Moreover, in response to salt stress, the phenylpropanoid biosynthesis pathway is stimulated, generating compounds with strong antioxidant potential [5,6,7,8,9,10,11,12,13,14,15,16,17,18,19,20]. Phenylpropanoids include flavonoids, lignin, phenolic acids, stilbenes, hydrolysable tannins, monolignols, lignans, and coumarins [18,19,20]. Lignin biosynthesis is a very complex network that is divided into three processes: (i) biosynthesis of lignin monomers; (ii) transport; and (iii) polymerization [15,18,20]. The main precursor of all phenylpropanoids is L-phenylalanine (L-Phe). Phenylalanine ammonia lyase (PAL) converts L-Phe to trans-cinnamic acid [5,12,13]. Tyrosine ammonia lyase (TAL) converts L-tyrosine to p-coumaric acid, which is involved in the work of the phenylpropanoid pathway [19,20,21,22,23,24,25]. A total of 37 *TaPAL* gene family members have been identified in wheat. The activity of these genes is regulated by various stresses that cause moisture deficiency, including salinity [22]. The biosynthesis of monolignols from trans-cinnamic acid and p-coumaric acid is regulated by the genes *C4H*—cinnamate 4-hydroxylase; *4CL*—4-coumarate—CoA ligase; *C3H*—p-coumarate 3-hydroxylase; *HCT*—p-hydroxycinnamoyl-CoA quinate/shikimate p-hydroxycinnamoyl transferase; *CCoAOMT*—caffeoyl-CoA O-methyltransferase; *CCR*—cinnamoyl-CoA reductase; *F5H*—ferulate 5-hydroxylase; *COMT*—caffeic acid O-methyltransferase; and *CAD*—cinnamyl alcohol dehydrogenase [23,24,25,26]. The regulation of the activity of genes involved in the biosynthesis of the main phenylpropanoid of the wheat cell wall, lignin, is not a well studied area of research. For example, waterlogging of wheat plants reduced the internode lignin content, and this effect was accompanied by transcriptional repression of the genes *PAL6*, *CCR2*, and *F5H2* and decreased activity of PAL [23]. The transcriptional activity of lignin synthesis genes in wheat has been shown to be regulated by irradiation with white, red, and blue LED lights [27] and salinity [19].

After a series of steps involving deamination, hydroxylation, methylation, and reduction, lignin monomers are produced in the cytoplasm and transported to the apoplast. Finally, lignin is generally polymerized from three main types of monolignols (sinapyl alcohol, S unit; coniferyl alcohol, G unit; and p-coumaryl alcohol, H unit) by peroxidase (POD) in the secondary cell wall [28]. PODs involved in lignification are relegated to class III PRX. Class III *PRXs* possess two distinct reaction mechanisms: a peroxidative cycle that uses H_2_O_2_ or other peroxides to oxidize their substrate (i.e., facilitating lignin polymerization by oxidizing monomers) and a hydroxylic cycle that converts H_2_O_2_ into other types of reactive oxygen species (ROS) [9,23,29]. Lignin can also be actively involved in the response to various abiotic stresses, including salinity [9,11,15]. In wheat roots, salt stress leads to considerable thickening of the cell walls in root vascular tissue [5,7,11].

Salicylic acid (SA) is also a product of the phenylpropanoid pathway [18,20]. SA is a phenolic phytohormone that plays a multifaceted signaling role in mediating plant growth, development, and defense against environmental stresses [30,31,32,33,34]. SA plays an indispensable role in reducing stress-induced ROS accumulation and regulating antioxidant enzymes. This means that SA is a strong antioxidant [14,35]. The amount of SA in different types of plants varies from 0.1 to 10 µg g^−1^ fresh weight [31]. SA is an important signal for the establishment of SAR (systemic acquired resistance), especially during short-term exposure to light after infection with a pathogen, and is considered to be a long-distance signaling molecule through phloem translocation, moving from affected to unaffected leaves [36]. SAR is an induced defense mechanism, the activation of which is accompanied by endogenous SA accumulation and the expression of pathogenesis-related (*PR*) genes. The *PR1* gene, a biomarker of SA-driven defense reactions, plays an important role in the regulation and protection of plants against a variety of biotic and abiotic stresses [35,37,38]. In wheat, *PR1* expression is induced by freezing, salinity, and osmotic stress [36,37,38].

It should be emphasized that all defense mechanisms in plants are integrated and triggered (regulated) by the action of signaling molecules. It is known that nitric oxide (NO) works as a signaling molecule and improves plant tolerance to salt stress by regulating the antioxidant and hormonal systems [11,16,39,40,41,42,43,44,45,46]. For a long time, NO was considered to be a gasotransmitter, as the smallest diatomic gas (30.006 g moL^−1^) Today, it is positioned as a gaseous phytohormone [35,42]. NO is involved in several plant growth processes, such as germination, root/leaf development (primary and lateral root growth), respiration, photosynthesis, flowering, fruit ripening, senescence, and pollen tube growth [39,41,45,46]. As a free radical gaseous signaling molecule, NO improves plant tolerance to major stresses such as salinity [10,42,46].

The growth-stimulating and protective effects of NO under various environmental stresses have been demonstrated in many plants [39,40], including wheat [12,43,44]. Even low (µM and nM) levels of NO can confer plant tolerance to a range of stresses, including salinity [12]. NO treatment enhances photosynthesis and photosystem efficiency, the transpiration rate and relative water content, phytohormones, non-enzymatic antioxidants, osmoprotectants, and proteins in plants under stress. Furthermore, it has been evidenced that NO can improve resistance to plant diseases by activating the expression of the *PR1* gene and inducing endogenous SA [47]. Fumigation with NO not only activated POD and PAL but also increased the lignin content in musk melon, which decreased the incidence of black spot disease [40]. One of the most common ways to study NO-mediated effects in plants is through the exogenous application of chemical donors, such as a sodium nitroprusside (SNP). This donor is widely used to study the role of NO in the life and resistance of plants [12,16,35,39,40,41,42,43,44,45,46,47,48,49,50,51]. It was found that SNP treatment caused PAL activation in pelargonium plants [41], in roots of soybean seedlings [16] and in the hypocotyls of *Vigna radiate* [45]. Under UV-B, exogenous NO activated additional PAL in the *Ginkgo biloba* callus. The use of cPTIO (2-(4-carboxyphenyl)-4,4,5,5-tetramethylimidazoline-1-oxyl-3-oxide), as a NO-specific scavenger, reduced PAL activation and prevented the development of NO-induced resistance [46]. As such, NO is able to regulate the components of the phenylpropanoid pathway. However, at the moment, there are practically no data on the effect of NO on the accumulation of lignin and its contribution to the implementation of plant protection mechanisms under stress.

This study aimed to analyze the effects of exogenous SNP (a donor of NO) on the state of the components of phenylpropanoid metabolism (i.e., lignin and SA) and its relationship with wheat seedling growth under normal and salinity (2% NaCl) conditions.

## 2. Results

### 2.1. Influence of SNP and cPTIO on Growth Parameters and Endogenous NO Content in Wheat

**Figure 1:**
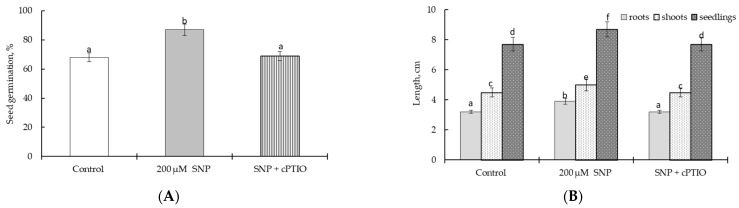
Effects of 200 μM SNP on seed germination percentage (**A**) and lengths of roots, shoots, and whole seedlings (**B**). The seeds were grown for four days on filter paper moistened with water (Control), 200 μM SNP (SNP), and a combination of 200 μM SNP and 100 µM cPTIO, a scavenger of NO (SNP + cPTIO). Seed germination percentage and length of seedlings were assessed on the 4th day of ontogeny. The variants in the same column marked with different letters represent mean values that are statistically different from each other according to Duncan’s test (n = 100, *p* ≤ 0.05).

The results revealed that the germination percentage of wheat seeds in the presence of 200 μM SNP was higher (by 128%) than the control (Figure 1A). Along with this, the presence of 200 µM SNP in the germination medium led to an increase in the lengths of roots (by 120%), shoots (by 110%), and whole seedlings (by 115%) (Figure 1B). However, the application of NO scavenger (cPTIO) prevented the ability of SNP to increase the seed germination percentage (Figure 1A) and length of seedlings (Figure 1B).

**Figure 2:**
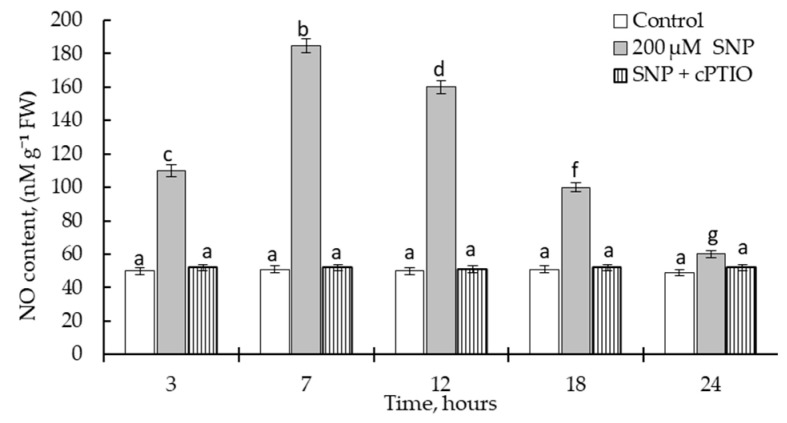
Effect of 200 μM SNP on endogenous nitric oxide (NO) generation in roots of wheat seedlings. Three-day-old seedlings were incubated for 24 h in media containing water (Control), 200 μM SNP (SNP), and a combination of 200 μM SNP and 100 μM cPTIO (SNP + cPTIO). The variants in the same column marked with different letters represent mean values that are statistically different from each other according to Duncan’s test (n = 30, *p* ≤ 0.05).

The results showed that exogenous 200 µM SNP application caused endogenous NO production in the roots of wheat seedlings (Figure 2). Particularly, incubation of 3-day-old seedlings in SNP solution for 7 h significantly increased (by 400%) the content of endogenous NO in the roots compared with the control seedlings. By 24 h of SNP exposure, the level of endogenous NO significantly decreased but was higher than in the control (Figure 2). The addition of NO scavenger (cPTIO) in the seedlings’ growth solution prevented SNP-induced endogenous NO generation (Figure 2).

Thus, these results indicated that the constant presence of an aqueous solution of 200 µM SNP in the germination medium significantly improved the physiological status of wheat and could be used as a donor of NO to improve plant growth.

### 2.2. Exogenous SNP Improves Wheat Plant Growth under Normal and Salinity Conditions

**Figure 3:**
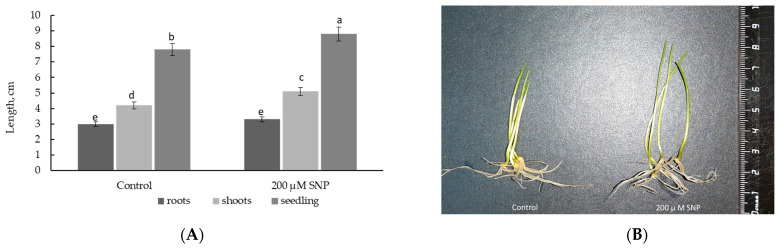
Effects of 200 μM SNP on the lengths of roots, shoots, and whole seedlings (**A**) and visual appearance of 4-day-old wheat seedlings (**B**). Three-day-old seedlings were incubated for 24 h in medium containing 200 μM SNP. The variants in the same column marked with different letters represent mean values that are statistically different from each other according to Duncan’s test (n = 30, *p* ≤ 0.05).

The growth rate, represented by the lengths of vegetative organs (roots, shoots, seedlings), is an integral indicator of the physiological state of the plant as well as a marker of the effectiveness of the use of various substances and the degree of exposure to various stress factors. The incubation of 3-day-old seedlings in 200 μM SNP for 24 h resulted in an increase in the lengths of roots (by 112%), shoots (by 121%), and whole seedlings (by 113%) relative to the control values (Figure 3A). Such growth-simulating action was evidenced by the visual appearance of these seedlings (Figure 3B).

**Figure 4:**
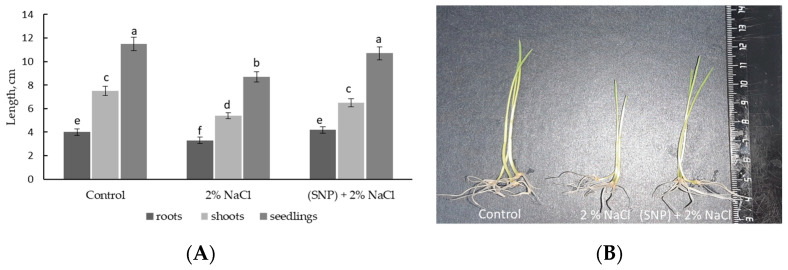
Effects of pretreatment with 200 μM SNP on the lengths of roots, shoots, and whole seedlings (**A**) and visual appearance of 5-day-old wheat seedlings (**B**) under salinity conditions (2% NaCl). Three-day-old seedlings were incubated for 24 h in medium containing 200 μM SNP, then exposed to 2% NaCl for 24 h. The variants in the same column marked with different letters represent mean values that are statistically different from each other according to Duncan’s test (n = 30, *p* ≤ 0.05).

The results revealed that exposure to salt stress over 24 h led to inhibited growth of roots, shoots, and whole seedlings by 82%, 63%, and 76%, respectively, relative to control levels (Figure 4A). The damaging effect of salinity on growth could also be observed by the visual appearance of these plants (Figure 4B). Pretreatment with SNP prevented stress-induced inhibited growth of roots, shoots, and seedlings in 5-day-old wheat and maintained wheat growth to control levels (Figure 4). Additionally, SNP-pretreated seedlings were characterized with better fresh and dry biomass under both normal and stress conditions (data not presented).

### 2.3. Effect of SNP Treatment on PAL and TAL Activities in Wheat Roots under Normal Conditions

**Figure 5:**
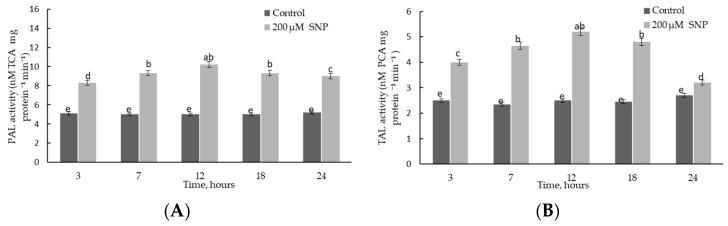
The effects of 200 µM SNP on the activities of PAL (**A**) and TAL (**B**) enzymes in the roots of 4-day-old wheat seedlings. Results are presented as mean ± SE (n = 6). Columns of each histogram marked with different letters represent mean values that are statistically different from each other according to Duncan’s test (*p* ≤ 0.05).

An analysis of the activities of PAL and TAL enzymes in wheat plants during incubation in the solution of 200 μM SNP is shown in Figure 5. It was revealed that SNP caused more than two times the transient activation of PAL (Figure 5A) and TAL (Figure 5B), with a maximum at 12 h of incubation. It should be noted that after 3 h of incubation in SNP solution, the activities of PAL and TAL enzymes increased up to 1.6 times relative to the control.

### 2.4. Effect of SNP on the Dynamics of POD Activity in the Roots of Wheat Seedlings and Lignin Content and Deposition in the Cell Walls of the Basal Part of the Roots

**Figure 6:**
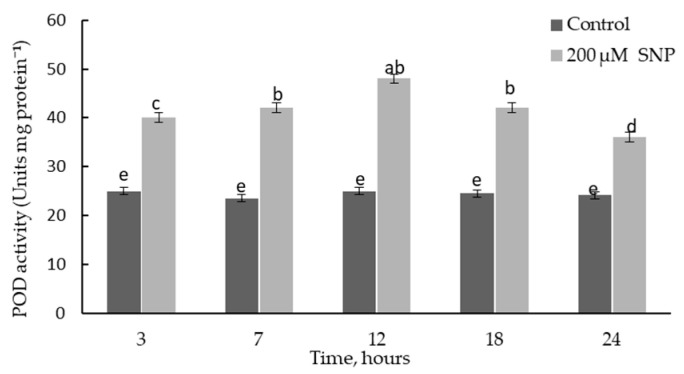
Activity of POD in roots of 4-day-old wheat plants incubated in medium with 200 µM SNP. Results are presented as mean ± SE (n = 6). Columns of each histogram marked with different letters represent mean values that are statistically different from each other according to Duncan’s test (*p* ≤ 0.05).

There are two principal stages in the synthesis of lignin structures: the biosynthesis of monolignol initiated by PAL and the polymerization of monolignol due to free radical coupling. The latter is characterized by the work of redox-active enzymes, particularly POD. During incubation of wheat seedlings in 200 µM SNP solution, the transient activation of POD was more than 1.8 times higher than in the control (Figure 6). After 3 h of exposure, the POD activity of SNP-treated plants was about 1.6 times higher than in the control.

Since PAL and POD are involved in lignin synthesis, it was important to compare the deposition of lignin in the cell walls of the basal part of wheat roots untreated and pretreated with SNP. The results showed that treatment of wheat seedlings with SNP for 24 h accelerated the lignification of the root cell walls compared to the control (Table 1). This was confirmed by the results obtained when quantifying the lignin content.

### 2.5. Gene Expression

**Figure 7:**
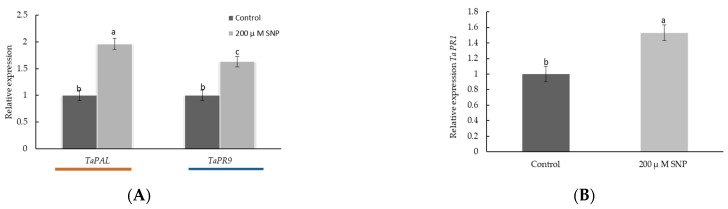
Influence of NO donor (SNP) on the relative expression of *TaPAL* and *TaPR9* genes in roots (**A**) and *TaPR1* gene (**B**) in seedlings of wheat. Three-day-old wheat seedlings were incubated in 200 µM SNP for 24 h. Expression values were normalized to the housekeeping gene *TaRLI* as an internal reference and expressed relative to the normalized expression levels in control plants at 0 pai. Results are presented as mean ± SE (n = 5). Columns of each histogram marked with different letters represent mean values that are statistically different from each other according to Duncan’s test (*p* ≤ 0.05).

To better understand the role of NO in the lignification process and biosynthesis of SA as well as its participation in the development of plant resistance to stresses, we analyzed the influence of 200 µM SNP on the transcriptional activity of the *TaPAL* gene encoding PAL, the *TaPR9* gene encoding anionic peroxidase (*TaPRX)*, as well as *TaPR1* gene expression, which is a marker of the SA signaling pathway. The results showed that the levels of transcripts of the *TaPAL* and *TaPR9* genes increased about 2 times and 1.6 times, respectively, in the roots of wheat seedlings treated with SNP (Figure 7A). Additionally, it was revealed that treatment with SNP increased the transcriptional activity of the *TaPR1* gene in the seedlings by 1.5 times (Figure 7B).

### 2.6. Dynamics of Endogenous SA Content in Wheat under the Action of 200 µM SNP

**Figure 8:**
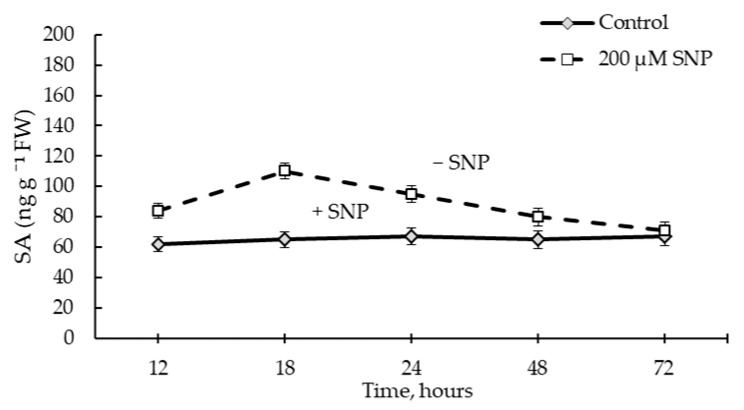
Changes in the endogenous salicylic acid (SA) content in wheat seedlings. + SNP—3-day-old seedlings incubated for 24 h in a solution of 200 μM SNP: − SNP—4-day-old seedlings pretreated with SNP for 24 h and transplanted into 1.5% sucrose for further growth up to 6 days. Results are presented as mean ± SE (n = 5).

Figure 8 shows the results of assessing the endogenous SA content in wheat seedlings exposed to 200 µM SNP for 24 h and the SA content in plants after transferring these seedlings to a solution of 1.5% sucrose. We believe that such an assessment of SA content allowed us to understand the role of SA more accurately in terms of the physiological effect of SNP on wheat plants.

During the period of incubation of plants in SNP, a transient accumulation of SA was found, the maximum of which occurred at 18 h of incubation (Figure 8). The SA content at this point was 1.6 times higher than in the control. Additionally, at 12 h of incubation, the SA content was 1.3 times higher than in the control. By 24 h of incubation, the SA content decreased, but it was 1.4 times higher than the control level.

After the removal of SNP from the incubation medium, the level of SA was maintained for a long time, and by day 6 of ontogenesis, the level of SA in plants was the same as in control seedlings (Figure 8).

### 2.7. PAL and TAL Activities in SNP-Pretreated Wheat Roots under Salinity Conditions

**Figure 9:**
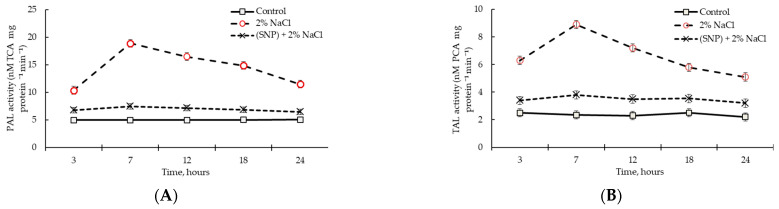
Dynamics of PAL (**A**) and TAL (**B**) activities in the roots of 200 µM SNP-pretreated plants under salinity conditions. Three-day-old seedlings were incubated for 24 h in medium containing 200 μM SNP, then exposed to 2% NaCl for 24 h. Results are presented as mean ± SE (n = 6).

Salinity caused transient activation of PAL (Figure 9A) and TAL (Figure 9B) at almost 3 times higher levels than under control conditions, with a maximum at 7 h of stress exposure. It should be noted that the activities of both enzymes had already increased by 2 times at 3 h of stress. Until the end of the stress exposure (24 h), the activities of PAL and TAL remained 2 times higher than their activities in control plants (Figure 9). Pretreatment with SNP led to a significant decrease in stress-induced activation of TAL and PAL enzymes (Figure 9).

### 2.8. Effect of 200 µM SNP on the Dynamics of POD Activity and Lignin Content and Deposition in the Cell Walls of the Basal Part of the Roots under Salinity Conditions

**Figure 10:**
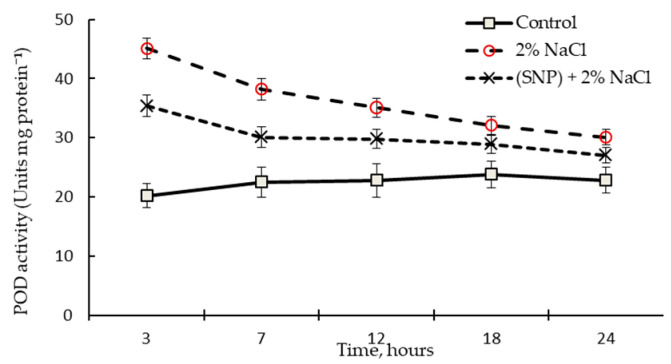
POD activity in the roots of 200µM SNP-pretreated and untreated 5-day-old wheat plants under salinity conditions. Three-day-old seedlings were incubated for 24 h in medium containing 200 μM SNP, then exposed to 2% NaCl for 24 h. Results are presented as mean ± SE (n = 6).

Salinity leads to the accumulation of ROS and activation of antioxidant enzymes. Therefore, it was not surprising that exposure to 2% NaCl for 3 h led to a more than 2-fold increase in POD activity compared to the control (Figure 10). After 24 h of salinity exposure, the activity of POD was 120% higher than in the control non-stressed plants (Figure 10). Pretreatment with SNP led to a significant decrease in stress-induced POD activity. At the beginning of the stress, the activity of POD in SNP-pretreated roots was about 1.7 times higher than in the control, while by 24 h of stress exposure, it was about 1.2 times higher than in the control.

It was found that exposure to 2% NaCl for 24 h led to a significant accumulation of lignin in the roots (Table 2). This was evidenced by the quantitative assessment of lignin in the roots, showing more intense staining of the cells of the basal part of the roots with phloroglucinol. It should be noted that lignification of the wheat roots was observed only in 6-day-old plants under normal conditions, and during the study period (5 days), lignin was not found in the roots of the control seedlings (Table 2).

### 2.9. Effect of 200 µM SNP and Salinity on the Endogenous SA Content in Wheat Plants

**Figure 11:**
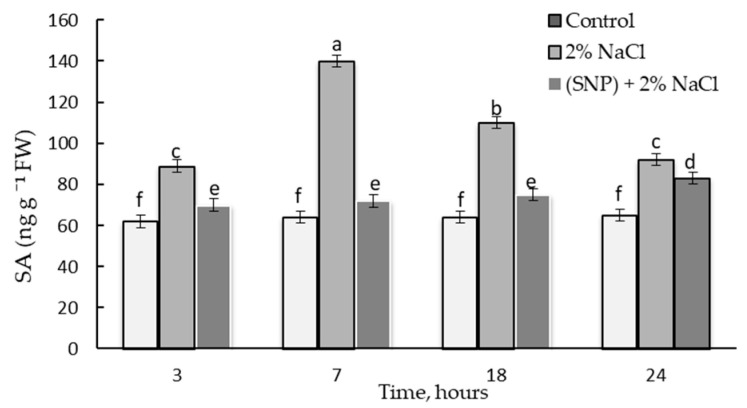
Influence of 200 µM SNP on the endogenous SA content in wheat plants under salinity conditions. Three-day-old seedlings were incubated for 24 h in medium containing 200 μM SNP, then exposed to 2% NaCl for 24 h. Results are presented as mean ± SE (n = 5). Columns of each histogram marked with different letters represent mean values that are statistically different from each other according to Duncan’s test (*p* ≤ 0.05).

In stressed wheat seedlings, a more than a two-fold endogenous SA accumulation was found compared to the control (Figure 11). Salinity caused a two-fold transitory accumulation of SA at 7 h of stress, but by 24 h of stress exposure, the level of SA had somewhat decreased and was 1.4 times higher than that in control seedlings. Pretreatment with NO maintained the higher level of endogenous SA under stress conditions, which was 1.1 times higher by 3 h of stress and 1.2 times higher by 24 h of stress (Figure 11).

## 3. Discussion

As a donor of NO, SNP is widely used to study the effects of NO on plants [39,40,41,42,43,44,45,46,47,48,49,50,51]. Chemically, it is an inorganic molecule composed of Fe (II) and NO+, being a derivate from iron-nitrosyl compounds. In solution, SNP releases NO+, iron (Fe (II)), and cyanide (CN−), which can sometimes mask the effects of NO [41,49]. In our study, we conducted experiments to evaluate the effects of 200 µM SNP on wheat plants using a technique of seedling incubation. Therefore, an important stage of the work was the evaluation of the effectiveness of 200 μM SNP as a donor of NO to improve wheat growth and its possible toxic effect on plants due to SNP-induced release of Fe (II) and CN−. NO is well known to increase seed germination and plant growth [10,16,35,39,43,45,47]. It was found that the presence of 200 μM SNP had a significant growth-stimulating effect, as evidenced by the seed germination percentage and seedling length data (Figure 1). The fact that the observed stimulation of plant growth was associated with SNP-induced NO was confirmed by experiments using cPTIO as a NO scavenger. This suggested that the constant presence of an aqueous solution of 200 µM SNP in the germination medium significantly improved the physiological status of wheat plants. The possible negative effects of Fe (II) and CN−, in our opinion, were compensated by the persistent significant accumulation of NO in the wheat roots induced by SNP (Figure 2). Additionally, this may have been due to the fact that the small concentration of SNP produced only a slight accumulation of Fe (II) and CN−; we did not observe any negative effect of 200 µM SNP on wheat growth (Figure 1). The use of cPTIO completely removed the accumulation of endogenous NO caused by the exogenous SNP (Figure 2). Thus, the obtained data testified to the fact that exogenous SNP had a growth-stimulating effect on wheat plants and caused a stable endogenous NO accumulation. These results supported the use of 200 µM SNP as a NO donor in our work.

It was found that incubation of 3-day-old seedlings in the solution of SNP for 24 h stimulated plant growth (i.e., lengths of roots and shoots) (Figure 3). Growth inhibition is one of the main plant responses to salinity stress [4,7,9,45]. Our data also demonstrated the significant growth inhibition of wheat seedlings after exposure to 2% NaCl for 24 h (Figure 4A). Pretreatment with SNP had a prolonged growth-stimulating effect on wheat. Although SNP did not prevent the negative effects of salinity stress on wheat seedlings, it helped to maintain the growth rate at least to the level of the control plants (Figure 4). It can be concluded that SNP pretreatment prevented the inhibition of growth processes caused by salinity and contributed to the preservation of the pronounced growth potential of the plants under stress conditions.

The principal ability of NO to regulate the lignification process and SA content in plants was previously studied [16,40,41,45,46]. An important step in understanding the involvement of NO in the regulation of the phenylpropanoid pathway was to analyze the dynamics of the activities of PAL and TAL in wheat roots. The activity of TAL under normal conditions was almost two times lower than that of PAL (Figure 5), which was consistent with the literature data [41]. Recent studies have discovered that TAL contributes to the typical characteristics of grass cell walls and accounts for about 50% of all lignin synthesized by the plant [52]. Exogenous application of NO induced activation of PAL and TAL—key enzymes of monolignol components that are further involved in the lignification [9,11,15] of wheat roots (Figure 5 and Figure 7A). An increase in the level of *TaPAL* gene transcript accumulation confirmed the involvement of the key enzymes of the phenylpropanoid pathway in the NO-mediated pathway.

SNP caused an increase in the activity POD and *TaPR9* (anionic class III *PRX*) gene transcript levels in wheat roots (Figure 6 and Figure 7A). It was shown that the specific activity of class III *PRX* was involved in the regulation of cell growth and differentiation in various tissues through the lignification of cell walls [53]. Increased POD activity may indicate an increase in the antioxidant status of the plant and its resistance [54,55]. Thus, NO caused a cascade of events that led to the accumulation of lignin in the SNP-treated plants, whereas lignin was not detected in the control plants (Table 1). In addition to the accumulation of lignin, a cascade of NO-mediated reactions caused the accumulation of endogenous SA (Figure 8). It should be noted that the parallel accumulation of lignin, SA, and an increase in the activity of POD under the influence of NO is a key moment that leads to an increase in the antioxidant status of plants (Table 1, Figure 6 and Figure 8). SA is an antioxidant and is able to regulate the activity of POD, as was confirmed by the literature data [56,57]. Prolonged maintenance of SA in wheat plants (up to 6 days of ontogeny) (Figure 6) probably makes an important contribution to the growth-stimulating and protective effects of NO (Figure 3 and Figure 4 and Figure 7B). SA is known to have a growth-stimulating effect on wheat plants. For example, Bagautdinova et al. showed the dose-dependent effect of SA on the growth and development of roots under normal and stress conditions [58]. The expression of the *TaPR1* gene was induced by several signaling molecules, including SA [24]. These data may indicate the ability of NO to regulate the accumulation of SA and the implementation of its effects through the accumulation of *PR* proteins associated with resistance.

An increase in the activities of PAL and TAL (Figure 9) enzymes was quite expected when the water regime was disturbed and this finding was consistent with the literature data [13,17]. This was accompanied by an increase in the activity of POD (Figure 10), which led to a significant accumulation of lignin in the roots of the plants (Table 2). Lignin biosynthesis plays a critical role in the adaptation of plants to salt stress [5,11]. Salt stress induces changes in the wall composition of specific root cell types, including the increased deposition of lignin and suberin in endodermal and exodermal cells. These changes can benefit the plant by preventing water loss and altering ion transport pathways [7]. SA overproduction via enhanced activity of the SA biosynthesis pathway enzyme PAL helps protect plants against environmental stresses [56,58]. This is consistent with our results: stress-induced PAL activation (Figure 9) led to a significant SA accumulation (Figure 11).

Strengthening of the barrier properties of cell walls during exposure to 200 µM SNP (Table 1) was an important defense mechanism against subsequent salinity exposure. This was quite understandable since the accumulation of lignin is an irreversible process. In addition to the stress level, the accumulation of lignin had a protective effect against salinity stress (Table 2 and Figure 4). Thus, the decrease in stress-induced activation of PAL, TAL (Figure 9), and POD (Figure 10) indicated the contribution of NO to the enhancement of antioxidant defense in wheat plants. Therefore, under salinity conditions, there was a significant stabilization of the antioxidant status, i.e., the SA content was at the control level (Figure 11) and the stress-induced POD activity was significantly decreased (Figure 10). This was also evidenced by the reduced negative effects of stress on plant growth under salinity conditions (Figure 4).

## 4. Materials and Methods

### 4.1. Plant Material, Seed Treatment, and Growth Conditions

The experiments were carried out on soft spring wheat seedlings (*Triticum aestivum* L., BBAADD 2n = 42, ‘Salavat Yulaev’). Wheat seeds were sterilized with 96% ethanol for 60 sec and germinated on filter paper moistened with water under the illumination of 200 μmoL m^2^ s^−1^ and a long-day photoperiod (16 h light/8 h dark) at a temperature of 22–23 °C. Sodium nitroprusside [SNP: (Na_2_[Fe(CN)_5_NO] 2H_2_O] was used as a nitric oxide donor at a concentration of 200 μM (this concentration of SNP was selected in our previous work) [43].

### 4.2. Design of Experiments

In the first group of experiments, wheat seeds (n = 100, 3 replicates) were grown for four days on filter paper moistened with water (Control), 200 μM SNP, and SNP + cPTIO to assess the growth-stimulating effect of SNP. Every day, transplantation was carried out in fresh solutions [43]. To confirm the participation of NO in SNP-induced reactions on wheat plants, we used 100 µM cPTIO (2-(4-carboxyphenyl)-4,4,5,5-tetramethylimidazoline-1-oxyl-3-oxide) as a scavenger of NO [12,16,35]. Preliminary experiments showed that cPTIO did not inhibit the growth of wheat plants and did not affect basic salt tolerance. On the 4th day of ontogeny, seed germination percentage and the lengths of roots, shoots, and seedlings were evaluated.

In the second group of experiments, 3-day-old seedlings were isolated from the endosperm, then parts of the plants were incubated in 1.5% sucrose (control) and in a solution of 1.5% sucrose containing 200 μM SNP for 24 h. Plant samples (root, shoots, or whole seedlings) were taken after 3, 7, 12, 18, and 24 h of SNP exposure.

In the third group of experiments, 3-day-old seedlings were isolated from the endosperm, then some of the seedlings were incubated for 24 h in a solution of 1.5% sucrose (control) and in a solution of 1.5% sucrose containing 200 μM SNP for 24 h. Then, SNP-pretreated and untreated 4-day-old seedlings were exposed to 2% sodium chloride (2% NaCl) for different time periods, and physiological, biochemical, and molecular parameters were further assessed. Seedlings incubated in 1.5% sucrose served as the controls in all experimental variants. Plant samples (root, shoots, or whole seedlings) were taken after 3, 7, 12, 18, and 24 h of stress exposure (depending on the purpose).

### 4.3. Determination of Plant Growth Parameters

The intensity of growth processes (seed germination, lengths of roots and shoots) was assessed according to [59].

### 4.4. Assays of Enzyme Activities

#### 4.4.1. PAL and TAL Activities

Protein extractions and kinetic assays were carried out according to the methods described by Jiang et al. [60] and Rösler et al. [61] with some modifications. Plant material (2.0 g) was stored at −80 °C after flash-freezing using liquid nitrogen. Frozen samples were ground in a mortar with appropriate buffer solution until the tissue was totally macerated. The mixture to grind included 5 mL of 0.1 M borate buffer containing 10% (*w*/*v*) polyvinylpolypyrrolidone (PVPP), 1 mM EDTA, and 50 mM β-mercaptoethanol. PAL activity was quantified by the production of trans-cinnamic acid (TCA), which was determined by measuring the absorbance at 290 nm [60] every minute up to 20 min at 37 °C using a spectrophotometer (UV-2800 Unico, United Products & Instruments, NJ, USA). The mixture contained 61 mM l-phenylalanine (dissolved in 0.1 M borate buffer), 30 mM sodium borate buffer (pH 8.8), and 75 µL of plant extract (60 mg mL^−1^), making the total volume of the resultant mixture 1 mL. L-phenylalanine (substrate) was added after a 10 min pre-incubation at 37 °C. Plant extract previously incubated in buffer without substrate was used as the blank solution. One unit (U) of PAL was defined as the amount of enzyme that caused an increase of 0.01 in absorbance at 290 nm per min. PAL activity was expressed as nM TCA mg protein^−1^ min^−1^. TAL activity was quantified in a similar manner by monitoring the rise of the p-coumaric acid PCA level by measuring the absorbance at 310 nm [60]. TAL activity was determined as nM PCA mg protein^−1^ min^−1^. Substrates of l-phenylalanine and l-tyrosine and products of trans-cinnamic acid (TCA) and p-coumaric acid (PCA) were used as standards, respectively.

#### 4.4.2. Peroxidase (POD) Activity

The activity of guaiacol peroxidase (EC 1.11.1.6) was evaluated according to [62]. Plant material (50–100 mg) was triturated with 10–15 mL of 0.01 M phosphate buffer (pH 6.0–6.2). The ground mass was well mixed and left for 30 min in the refrigerator. Then, the homogenate was centrifuged for 15 min at 14,000 rpm in a MDX-310 high-speed centrifuge (5415 K Eppendorf, Hamburg, Germany). POD activity was determined in aliquots of the supernatant by measuring the absorbance at 440 nm using a spectrophotometer (UV-2800 Unico, United Products & Instruments, NJ, USA). For the analysis of one biological sample, 2 mL of phosphate buffer, 0.5 mL of guaiacol solution, 0.5 mL of 0.03% hydrogen peroxide solution, and 10–50 µL of liquid supernatant were used. The time was noted with a stopwatch and the color development was monitored for 5 min. A cuvette with distilled water instead of the supernatant was used as the control. POD activity was determined as units mg protein^−1^.

Protein concentrations were determined using the Bradford method [63].

### 4.5. Lignin Determination

#### 4.5.1. Quantitative Determination of Lignin

Lignin was semi-quantitatively estimated by following the method described by Sancho et al. [64]. About 100 mg of root tissue was thoroughly rinsed in hot water, and after centrifugation, the insoluble particles were pelleted out and rinsed in 100% ethanol. The dry residue thus obtained was solubilized for 2.5 h in a solution of 2.5 mL of HCl/ethanol. Further, 10 μL of 20% phloroglucinol-HCl was mixed with 1 mL of the previous solution. After 30 min of incubation, the absorbance of the mixture was recorded at 540 nm using a spectrophotometer (UV-2800 Unico, United Products & Instruments, NJ, USA).

#### 4.5.2. Lignin Deposition In-Situ

Lignin deposition in the cell walls of the basal part of the roots was assessed histochemically. To achieve this, fresh hand-cut segments of the root basal parts of wheat treated or untreated with SNP for one day were assessed by visualization of the intensity of red-violet lignin staining with Wiesner reagent (phloroglucinol-HCl) [65]. Wiesner staining was performed by pouring a few drops of 5% phloroglucinol ethanol solution on the section, adding one drop of 30% HCl, and then covering the section with a cover slip.

### 4.6. Endogenous Salicylic Acid (SA) Assay

The total endogenous SA was analyzed by high-performance liquid chromatography (HPLC) [66]. The seedlings (0.2–0.3 g) were extracted using 20 mL of dH_2_O (90–100 °C) and incubated at 100 °C for 30 min with subsequent cooling. Membrane filters (0.45 µm) (Chromafil Xtra PTFE–45/13, Macherey-Nagel GmbH Co, Duren, Germany) were used to filter the extracts. The analysis was caried out using a Waters Breeze chromatograph (Waters Corporation, Milford, MA, USA) with a Waters 2487 Dual & Absorbance diode array detector at 305 nm. A Pursuit C18 column (250 × 4.6 mm, 5 µm) (Agilent Technologies, Santa Clara, CA, USA) was used. As a mobile phase, 0.5% H_3_PO_4_: acetonitrile = 65:35 (1.0 mL min^−1^) was used. A total of 20 µL of the extract was injected into the chromatography system using a Waters 2707 automated sampler (Waters Corporation, Milford, MA, USA). The software calibration curve was used to calculate the total SA content.

### 4.7. Endogenous NO Measurement

Plant tissues (0.5 g) were ground into a powder with a mortar and pestle and homogenized in 2 mL of ice-cold Na-acetate buffer (pH 3.6), followed by centrifugation at 10,000× *g* for 10 min at 25 °C. Then, 1 mL of extract and 1 mL of Greiss reagent were mixed and incubated at room temperature for 30 min. The absorbance of the nitrite- and nitrate-derived NO content was measured at 540 nm using a Smart Spec Plus spectrophotometer (Bio-Rad, Hercules, CA, USA). Endogenous NO content was calculated by comparison to a standard curve of NaNO_2_. NO content was expressed as nM g^−1^ FW [67].

### 4.8. Isolation of RNA and Performing the Quantitative Real-Time Polymerase Chain Reaction (qRT-PCR)

Whole plants and separated roots per biological replication were collected and fixed in liquid nitrogen after 24 h of SNP incubation. Total RNA was extracted using TRIzol™ Reagent (Merck KGaA, Sigma-Aldrich, Darmstadt, Germany) according to the manufacturer’s instructions. The potential contaminating DNA was digested with DNaseI (Synthol, Moscow, Russia). First-strand cDNA was synthesized using M-MLV reverse transcriptase (Synthol, Moscow, Russia). Oligo (dT)15 was used as a primer, and the reverse transcription reagents were incubated at 37 °C for 1 h in a total volume of 25 µL. After 10-fold dilution, 2 µL of the synthesized cDNA was used for qPCR. Quantitative PCR was performed by polymerase chain reaction in real time using a set of predefined reagents EvaGreenI (Synthol, Moscow, Russia) and the CFX Connect real-time PCR Detection System device (BioRad Laboratories, Hercules, CA, USA). The qPCR program was as follows: 95 °C for 5 min, 40 cycles of 95 °C for 15 s, 60 °C for 20 s, and 72 °C 30 s. After the final PCR cycle, a melting curve analysis was conducted to determine the specificity of the reaction (95 °C for 15 s, 60 °C for 1 min, and 95 °C for 15 s). Primers for qRT-PCR were designed using a web-based primer designing tool from IDT (http://eu.idtdna.com/Scitools/Applications/Primerquest, accessed on 5 November 2022) (USA). The sequences of all the primers are presented in Table 3. To standardize the data, the *TaRLI* (RNaseLinhibitor-like) wheat gene was used as an internal reference for the real-time qPCR analysis. The efficiency of each primer pair was determined using a 10-fold cDNA dilution series in order to reliably determine the fold change. The quantification of gene expression was performed using the CFX Connect real-time PCR Detection System (Bio-Rad Laboratories, Hercules, CA, USA). The relative gene expression was calculated using the delta-delta Ct method [68]. Three independent biological and three technical replicates were performed for each experiment.

### 4.9. Statistical Analysis

All physiological, biochemical, and molecular analyses were carried out with three biological and three analytical replicates. Root segments of ten plants were fixed for each treatment for histochemical localization of lignin deposition and content. Microphotographs represent the results of a typical variant from a series of experiments. Experimental data were expressed as mean ± SE, which were calculated in all treatments using MS Excel. The significance of differences was assessed by ANOVA followed by Duncan’s test (*p* ≤ 0.05) using STATISTICA 10.0 software.

## 5. Conclusions

Our results demonstrated that 200 μM SNP had a growth-stimulating and protective effects on wheat plants under salinity conditions. An important contribution to the implementation of these effects was the ability of SNP (a donor of NO) to positively regulate the activities of PAL, TAL and POD, the expression of *TaPAL* and *TaPRX* genes, as well as accelerate root lignification under normal growth conditions. Increased endogenous SA content, *TaPR1* gene expression, and antioxidant status were also revealed in plants. These events made an important contribution to the preadaptive effect of exogenous SNP on wheat plants under further stress. Under salinity conditions, SNP pretreatment decreased stress-induced PAL, TAL, and POD activities, endogenous SA generation, and resulted in additional lignification of root cell walls. Thus, the findings indicated that the components of the phenylpropanoid pathway (i.e., lignin and SA) were involved in the implementation of the growth-stimulating and protective effects of exogenous SNP in wheat plants under salinity stress.

## Figures and Tables

**Table 1:** Effect of SNP on the deposition and content of lignin in roots of 4-day-old wheat. Lignin deposition was determined in the cell walls of the basal part of the roots of 4-day-old seedlings. Three-day-old seedlings were incubated in 200 µM SNP. Bar = 100 µm.

Visual Assessment of Lignin Deposition in Roots	Lignin Content (ΔA 540 nm/g FW)
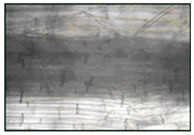 Control	0
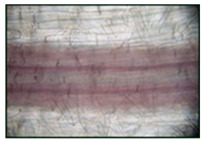 200 µM SNP	1.35 ± 0.062

**Table 2:** Effect of 200 µM SNP and salinity (2% NaCl) on the lignin content of roots of 5-day-old wheat plants. Three-day-old seedlings were incubated for 24 h in medium containing 200 μM SNP, then exposed to 2% NaCl for 24 h. Lignin deposition was determined in the cell walls of the basal part of the roots. Bar = 100 µm. The variants in the same column marked with different letters represent mean values that are statistically different from each other according to Duncan’s test (n = 10, *p* ≤ 0.05).

Visual Assessment of Lignin Deposition in Roots	Lignin Content in Roots (Δ A 540 nm/g FW)
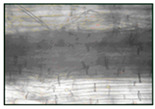 Control	0
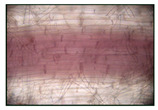 2% NaCl	1.92 ± 0.076 ^b^
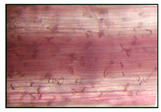 (SNP) + 2% NaCl	2.89 ± 0.115 ^a^

**Table 3:** Primers used for qRT-PCR.

Gene’s Product	Genes	GenBank Accession Number	Sequence (5′-3′)	
			Forward Primers	Reverse Primer
RNase L inhibitor	*TaRLI*	AY059462	ttgagcaactcatggaccag	gctttccaaggcacaaacat
Phenylalanine ammonia-lyase	*TaPAL*	X99725	ggcgtcaaaacatggcgtc	agtccgagaagtccgaga
*PR1*	*Ta PR1*	AF384143	ataacctcggcgtcttcatc	gcttattacggcattcctttt
Peroxidase, *PR9*	*TaPR9*	TC 151917	tcgacaagcagtactaccacaa	ccgaagtccgagaagaactg

## Data Availability

Not applicable.
